# A Frog-Derived Immunomodulatory Peptide Promotes Cutaneous Wound Healing by Regulating Cellular Response

**DOI:** 10.3389/fimmu.2019.02421

**Published:** 2019-10-17

**Authors:** Xiaoqin He, Yang Yang, Lixian Mu, Yandong Zhou, Yue Chen, Jing Wu, Yipeng Wang, Hailong Yang, Min Li, Wei Xu, Lin Wei

**Affiliations:** ^1^Jiangsu Key Laboratory of Infection and Immunity, Institutes of Biology and Medical Sciences, Soochow University, Suzhou, China; ^2^National Health Commission Key Laboratory of Parasitic Disease Control and Prevention, Jiangsu Provincial Key Laboratory on Parasite and Vector Control Technology, Jiangsu Institute of Parasitic Diseases, Wuxi, China; ^3^School of Basic Medical Sciences, Kunming Medical University, Kunming, China; ^4^Department of Pharmaceutical Sciences, College of Pharmaceutical Sciences, Soochow University, Suzhou, China

**Keywords:** immunomodulatory peptide, wound healing, amphibian, *Odorrana tormota*, skin

## Abstract

Wound healing-promoting peptides exhibit excellent therapeutic potential in regenerative medicine. However, amphibian-derived wound healing-promoting peptides and their mechanism of action remain to be further elucidated. We hereby characterized a wound healing-promoting peptide, Ot-WHP, derived from Chinese concave-eared frog *Odorrana tormota*. It efficiently promoted wound healing in a mouse model of full-thickness wounds. Ot-WHP significantly increased the number of neutrophils in wounds, and modestly promoted neutrophil phagocytosis and phorbol myristate acetate (PMA)-induced neutrophil extracellular trap formation. Ot-WHP also significantly increased the number of macrophages in wound sites, and directly induced chemokine, cytokine and growth factor production in macrophages by activating mitogen-activated protein kinases (MAPKs) and nuclear factor-κB (NF-κB) signaling pathways. Of note, Ot-WHP did not act as a chemoattractant for neutrophils and macrophages, suggesting its chemotactic activity depends on inducing chemoattractant production in macrophages. Besides, Ot-WHP directly promoted keratinocyte migration by enhancing integrin expression and cell adhesion. In addition, Ot-WHP significantly enhanced the cross-talk between macrophages and keratinocytes/fibroblasts by promoting keratinocyte/fibroblast proliferation, and fibroblast-to-myofibroblast transition despite having no direct effects on keratinocyte/fibroblast proliferation, and fibroblast differentiation. Collectively, Ot-WHP directly elicited the production of regulatory factors in macrophages, consequently initiated and accelerated the inflammatory phase by recruiting neutrophils and macrophages to wounds, and in turn enhanced the cross-talk between macrophages and keratinocytes/fibroblasts, additionally promoted keratinocyte migration, and finally promoted cutaneous wound healing. Our findings provide a promising immunomodulator for acute wound management and new clues for understanding the mechanism of action of amphibian-derived wound healing-promoting peptides.

## Introduction

Skin acts as the outer barrier that is challenged by a series of external stress factors, resulting in frequent cell and barrier damage (1). After injury, wound healing is essential to restore the integrity of the skin barrier (2). Although the restoration of a functional epidermal barrier is highly efficient in normal physiological conditions, the normal repair response will go awry when the injured skin does not repair in a timely manner, and in turn results in delayed healing, chronic wounds or abnormal scar formation (1, 2). A neglected fact is that traumatic injury has been one of the leading causes of mortality in many countries (1). In addition to trauma, there are millions of surgical wounds created in the course of routine medical care every year (3). In fact, the number of patients who are suffering from impaired healing conditions and chronic wounds is reaching epidemic proportions and will become even more burdensome in both human health and economic terms (3). Although a central concern of clinical care has focused on facilitating the healing process in clinical injuries, minimizing the aesthetic impact on the patient and maximal restoration of tissue function, we still lack efficient therapies for treating non-healing wounds, speeding up the repair of non-healing wounds, and speeding up the repair of acute wounds. Hence, there is a strong medical and social need to improve therapeutic approaches for enhancing the endogenous tissue regenerative capacity.

Cutaneous wound healing is a highly orchestrated biological process, requiring the collaborative efforts of many different cell types and cellular processes to achieve restoration of tissue integrity (4, 5). Normal cutaneous wound repair is characterized by distinct, yet overlapping phases of wound healing, termed hemostasis, inflammation, proliferation, and remodeling (6, 7). Molecular and cellular mechanisms investigation indicates that the spatiotemporal process of wound healing can be arbitrarily divided into three stages, including early stage, intermediate stage and late stage (1). Early stage includes hemostasis, activation of keratinocytes and recruitment of inflammatory cells. Intermediate stage involves proliferation and migration of keratinocytes, proliferation of fibroblasts, matrix deposition and angiogenesis. The late stage contains remodeling of extracellular matrix, scar formation and restoration of barrier (1, 2, 6, 8). The spatiotemporal process of wound healing is tightly controlled by multiple cell types that secrete numerous signaling molecules, such as cytokines, chemokines, and growth factors, to achieve wound closure and functional restoration of the skin barrier. All of these phases and signaling molecules are potential therapeutic targets for modulating wound healing progression. The past decade has witnessed some new developments of various biological active therapeutic attempts, including epidermal growth factor (EGF), fibroblast growth factor 2 (FGF-2), vascular endothelial growth factor (VEGF), platelet-derived growth factor (PDGF), keratinocyte growth factor-1 (KGF-1), granulocyte macrophage colony-stimulating factor (GM-CSF), and granulocyte colony-stimulating factor (G-CSF) and so on (1, 9). However, therapies based on growth factors have not yet been proven to be broadly effective in clinical application (10). Notably, these growth factors are large sizes that correspond to higher production cost which limit their widespread use in clinical. Recently, immunomodulatory peptides (also called wound healing-promoting peptides) with small size and potent wound-healing-promoting activities are becoming attractive candidates for treatment of wounds (11, 12).

Amphibian skins are effective natural barriers between the organism and the environment. They play key roles in defense, respiration, and water regulation (13). As an outer covering of the body, amphibian skins are susceptible to biotic or abiotic injuries, such as predation, parasitization, microorganism infection, and physical harm, including aseptic wounds and radiation (13). However, amphibians have a strong capacity to restore their skin injuries with no post-operative care (14). They have evolved an effective wound healing system including a variety of wound healing peptides (14, 15). Although several amphibian-derived wound healing-promoting peptides have been most recently investigated (13–24), the mechanism of action of these wound healing peptides, such as their effects on neutrophils, their effects on the cross-talk between effector cells, and their direct chemotactic effects on neutrophils and macrophages, remains to be further elucidated.

The Chinese concave-eared frog *Odorrana tormota* (formerly *Amolops tormotus*) is an arboreal, nocturnal frog that lives near noisy streams in Huangshan Hot Springs, China (25–29). Previous research primarily focused on the ultrasonic communication between male and female *O. tormota* during reproduction (25–29), but no *O. tormota*-derived bioactive peptides were investigated up until now. To find more amphibian-derived wound healing peptides and further understand their mechanism of action, the peptidomics of the skin secretions of *O. tormota* and their effects on wound healing were assayed *in vivo* and *in vitro*, and a *O. tormota*-derived wound healing peptide, designated as Ot-WHP, was identified from the skin of concave-eared frog. Ot-WHP showed potential wound-healing-promoting activity in a mouse model of dermal full-thickness wound. The effector cell types of Ot-WHP, the direct or indirect effects of Ot-WHP on effector cells and the effects of Ot-WHP on the crosstalk between different effector cells were investigated. Our results suggest that Ot-WHP acts as an efficient wound healing immunomodulator.

## Materials and Methods

### Animals and Ethics Approval

Adult *O. tormota* (both sexes, *n* = 30) were collected from Huangshan Hot Springs in Tangkou town (30°30′ N, 118°13′ E), China. Frogs were housed in a plastic box (56.3 cm × 42.5 cm × 32.3 cm), supplemented with a little water, and fed with mealworm larvae *Tenebrio molitor*. BALB/c mice (female, 18–20 g) were purchased from Shanghai Slac Animal Co. Inc. and housed in a pathogen-free facility. Animal experiments were performed in accordance with the Guide for the Care and Use of Medical Laboratory Animals (Ministry of Health, People's Republic of China, 1998), and were approved by the Animal Care and Use Committee as well as the Ethical Committee of Soochow University (SYXK2017-0043).

### Cells

Bone marrow-derived macrophages (BMDMs) were prepared according to our previous method (30). BMDMs isolated from the tibia and femur of BABL/c mice were cultured in RPMI 1640 medium with 2 mM glutamine, 10 ng/ml M-CSF (PeproTech, NJ, USA) for 5 days. Differentiated BMDMs were harvested and re-plated for the experiment. Bone marrow-derived neutrophils were prepared as described previously (31). Briefly, bone marrow from BABL/c mice was harvested, rinsed with 5 ml PBS, filtered through a cell strainer (70 micron), and centrifuged at 500 × *g* for 5 min. A PBS diluted Percoll gradient with 72, 64, and 54% layers was created, and the bone marrow-derived neutrophils pellet was re-suspended in PBS and over-layered onto this gradient. The Percoll gradient was centrifuged for 25 min at 950 × *g*. Neutrophils were collected from the 72%/64% interface, and washed with ACK lysing buffer for 5 min, followed by suspension in PBS and centrifuged at 500 × *g* for 5 min. Neutrophils were re-suspended in 5 ml RPMI 1640 supplemented 10% FBS for cell counts and experiment. THP-1 cells were cultured in RPMI 1640 containing 5 nM phorbol myristate acetate (PMA, Sigma, USA) for 24 h, and cells were washed three times with PBS after differentiation into macrophage-like cells (32). Keratinocytes (HaCaT) were cultured in DMEM (Hyclone, UT, USA). Fibroblasts from newborn BABL/c mouse skin were isolated and preserved according to our previous paper (33), and were cultured in DMEM (Hyclone, UT, USA). All cells were supplemented with 10% FBS (Hyclone, UT, USA) and 100 U-100 μg/ml penicillin-streptomycin (GIBCO, USA), and were cultured in a humidified incubator under 5% CO_2_ at 37°C.

### Synthetic Peptides

Synthetic peptides, including Ot-WHP, scrambled Ot-WHP and AH90, were purchased from Synpeptide Co. Ltd. (Shanghai, China), and analyzed by RP-HPLC and MALDI-TOF MS to ensure that the purity was higher than 98%.

### Isolation of Wound Healing-Promoting Peptide Candidate

Frog skin secretions were collected as previously described (34). Briefly, frogs were stimulated with anhydrous ether, and skin secretions were collected, centrifuged, and lyophilized. Lyophilized skin secretion sample was dissolved in 10 ml PBS (0.1 M, pH 6.0, total absorption of 10 ml skin secretion solution at OD280 is 520), and was centrifuged at 5,000 × *g* for 10 min. The supernatant was applied to a Sephadex G-50 (Superfine, Amersham Biosciences, 2.6 cm × 100 cm) gel filtration column, and eluted with PBS (0.1 M, pH 6.0) at a flow rate of 3.0 ml/10 min. Absorbance of the eluted fractions was monitored at 280 nm. Fractions with wound healing-promoting effect on full-thickness wounds in mice were pooled, and applied to a C18 reversed-phase high-performance liquid chromatography column (RP-HPLC, 5 μm particle size, 110 Å pore size, 250 mm × 4.6 mm, Gemini, CA, USA) twice, using a linear gradient of 0–60% acetonitrile supplemented with 0.1% (v/v) trifluoroacetic acid/water over 80 min. The eluted peptide (0.5 μl) was spotted onto a matrix-assisted laser desorption ionization time-of-flight (MALDI-TOF) plate with 0.5 μl α-cyano-4-hydroxycinnamic acid matrix (10 mg/ml in 60% acetonitrile) to confirm its purity. The purified peptide candidate was subjected to a pulsed liquid-phase Shimadzu protein sequencer (PPSQ-31A; Shimadzu, Kyoto, Japan), according to the manufacturer's instruction.

### cDNA Cloning

Total RNA extraction from the frog skin was performed using RNeasy Protect Mini Kit (Qiagen, Hilden, Germany) following the manufacturer's instruction. A SMART^TM^ PCR cDNA synthesis kit purchased from Clontech (Palo Alto, CA) was then used to construct the skin cDNA library, producing a library containing approximately 3.1 × 10^5^ independent colonies. A PCR-based method was used to clone and isolate the nucleotide sequence encoding Ot-WHP from the cDNA library (35). A sense primer (5' PCR primer, 5′-AAGCAGTGGTATCAACGCAGAGT-3′, provided by the cDNA library construction kit), and an antisense primer (S1(5′- CC(A/G)TG(A/C/G/T)GG(A/C/G/T)CC(A/C/G/T)A(A/G) (A/G)TCCCA-3′, designed from the amino acid sequence of Ot-WHP determined by Edman degradation) were used in the first step PCR reaction. The full length nucleotide sequence was cloned using another sense primer (S2, 5′-ATGTTCACCTTGAAGAAATTC-3′, designed from the nucleotide sequence obtained by the first step PCR reaction), and another antisense primer (3′ PCR primer, 5′-ATTCTAGAGGCCGAGGCGGCCGACATG-3′, provided by the cDNA library construction kit). PCR conditions were 2 min at 95°C, followed by 25 cycles of 10 s at 92°C, 30 s at 52°C, 30 s at 72°C, and concluded by 10 min extension at 72°C. The PCR products were cloned into pGEM-T Easy vector (Promega, Madison, WI, USA), and positive clones were selected for DNA sequencing performed by Genewiz Co. Ltd. (Suzhou, China).

### Full-Thickness Wound Model in Mice

BABL/c mice (female, 18–20 g, *n* = 6) were anesthetized with 2% sodium pentobarbital sodium (0.1 ml/20 g body weight, i.p.). After dorsal hairs were removed by an electric clipper, the naked skin was cleaned with betadine, and a full-thickness skin wound was created on the back of each mouse using an 8-mm-diameter biopsy punch, then each postsurgical mouse was caged individually until termination of the experiment (16, 18). Peptide or EGF samples were dissolved in PBS. Each wound was treated with vehicle (20 μl, PBS), synthetic Ot-WHP (20 μl, 200 μg/ml), natural Ot-WHP (20 μl, 200 μg/ml), scrambled Ot-WHP (20 μl, 200 μg/ml), AH90 (20 μl, 200 μg/ml), or EGF (20 μl, 100 μg/ml, epidermal growth factor) one time per day from days 0 to 8, respectively. Wound area was monitored by taking digital photographs at days 0, 2, 4, 6, and 8, and the wound area was obtained from the photographs using PhotoShop (Adobe Photoshop Element 2.0, Adobe Systems, San Jose, CA, USA). The wound healing rate of each wound at days 2, 4, 6, and 8 was the wound area of day 2/day 0 × 100%, day 4/day 0 × 100%, day 6/day 0 × 100%, and day 8/day 0 × 100%, respectively.

### Histology and Immunohistochemistry

At the indicated time points (days 0.5, 1, 2, 3, 4, 8) post injury, mice (*n* = 6) were sacrificed, and biopsy of wounds including healed tissues (about 8 mm in diameter) were taken, fixed in 10% formalin, dehydrated through an increasing concentration of ethanol, cleared in xylene, followed by embedding in paraffin wax. Biopsies were then sectioned into 5 μm slices using a histocut (Leica, RM2235, Germany) for hematoxylin and eosin (H&E) staining and immunohistochemistry analysis, respectively. All sections were deparaffinized and rehydrated.

For histological analysis, sections were stained with H&E. The changes, including width of the wound and thickness of the neoepithelium (NE) and granulation tissue (GT), were measured by IPLab imaging software (BD Biosciences, Bedford, MA, USA). A semi-quantitative score system was used to evaluate epidermal regeneration of all slices (16, 36). In this system, a 3-point scale (1, little; 2, moderate; 3, complete) was used to evaluate epidermal regeneration.

For immunohistochemistry, after routinely dewaxing, hydrating, rinsing and undergoing antigen repair, the sections were incubated with 5% bovine serum albumin (BSA) in PBS to block nonspecific binding of antibodies. Sections were then incubated with primary antibodies containing rat anti-Ly6G and anti-F4/80 (1:200, abcam, Cambridge, MA, USA), and rabbit anti-α-SMA (1:1000, abcam, Cambridge, MA, USA) primary antibody at 4°C overnight. Rat IgG (1:500) and rabbit IgG (1:500) were served as isotype controls. The immunoreactivity was visualized with a horseradish peroxidase-conjugated secondary antibody and 3,3-diaminobenzidine tetrachloride (DAB). Neutrophil or macrophage infiltration was evaluated by counting the cells immunostained with anti-Ly6G and anti-F4/80 antibody, respectively.

For collagen deposition assay, sections were stained with Masson's trichrome stain kit following the manufacturer's instruction (Polysciences, Warrington, PA, USA).

### Neutrophil Extracellular Traps (NETs) Assay

Neutrophil suspension (200 μl/well, 1 × 10^6^ cells/ml) was seeded into an 8 well-cover slip chamber in 2% FBS RPMI 1640. Peptide was added at a final concentration of 25, 50, and 100 μg/ml, a same volume of vehicle (PBS) served as negative control, and PMA (100 nM) served as positive control. After incubation for 4 h at 37°C, nuclei and NETs were visualized by stained with DAPI (Invitrogen, USA) or anti-Cit-H3, respectively. NETs were observed using a confocal microscope (×60, Nikon, Japan) (37).

### Phagocytosis Assay

*Staphylococcus aureus* (ATCC 25923) and *Escherichia coli* (ATCC 25922) were cultured in Luria-Bertani broth at 37°C to exponential phase, and preloaded with CFSE fluorescent dye (10 mM, dissolved in PBS) at 37 °C for 30 min. After incubation of bacteria with 1% paraformaldehyde in PBS at 37 °C for 1 h, bacteria were washed five times in fresh PBS. Neutrophils were pre-incubated with Ot-WHP (25, 50, 100 μg/ml) or a same volume of vehicle (PBS) for 1 h, and then cultured with the CFSE-labeled bacterial particles (multiplicity of infection = 100) for 1.5 h. Neutrophils were thoroughly washed with PBS, and the extracellular fluorescence was quenched with 15 mg/ml trypan blue in PBS. Neutrophils were analyzed by flow cytometry for CFSE fluorescence (38).

### Enzyme-Linked Immunosorbent Assay (ELISA)

BMDMs (5 × 10^5^ cells/well, in RPMI 1640, 2% FBS) were seeded in 24-well plates, and incubated with vehicle (PBS) or different concentrations of peptide (25, 50, 100 μg/ml) for 24 h. Supernatants were harvested and centrifuged at 10,000 × *g* for 10 min for detecting chemokines (including CXCL1, CXCL2, CXCL3, and CCL2), and cytokines (including TNF-α, IL-1β, IL-6, and TGF-β1).

Neutrophils (5 × 10^5^ cells/well, in RPMI 1640, 2% FBS) were seeded in 24-well plates, and incubated with vehicle (PBS) or different concentrations of peptide (25, 50, 100 μg/ml) for 4 h. Supernatants were harvested and centrifuged at 10,000 × *g* for 10 min to detect chemokines (CXCL1, CXCL2, CCL2) and cytokines (TNF-α and IL-1β).

Homogenates of the biopsy of wounds (days 0, 0.5, 1, 2, 4, and 6), including healed tissues (about 8 mm in diameter) from 6 mice, were prepared in 0.1 M PBS (containing 1 mM PMSF, 1 ml/g tissue) by a glass homogenizer. The homogenates were centrifuged at 12,000 × *g* for 30 min at 4°C. The supernatants were collected for detecting the level of chemokines (CXCL1 and CCL2) and cytokines (TNF-α and TGF-β1) in the wound sites.

All the chemokine and cytokine levels of different samples were detected by ELISA kits (eBioscience, California, USA) according to the kit instruction.

### Western Blot Analysis

Effects of Ot-WHP on MAPKs, NF-κB and Smad signal pathways in BMDMs were assayed by using several specific inhibitors and Western blot analysis. BMDMs (1 × 10^6^ cells/ml, 2 ml) were plated into a 6-well culture plate and transferred to serum-free DMEM for a 16 h incubation. For inhibitor assay, BMDMs were pre-incubated with ERK inhibitor (U0126, 10 μM), JNK inhibitor (SP600125, 10 μM), p38 inhibitor (SB203580, 10 μM), NF-κB inhibitor (BAY11-7082, 2 μM), PI3K inhibitor (LY294002, 10 μM) or a same volume of DMSO for 1 h, respectively, and then were stimulated with Ot-WHP (100 μg/ml) or a same volume of vehicle (PBS) for 24 h. The levels of CXCL1, CCL2, TNF-α, and TGF-β1 in the culture medium were detected by ELISA kits (eBioscience, USA). For MAPK and NF-κB signaling assay, BMDMs were incubated with Ot-WHP (0, 25, 50, 100 μg/ml) for 30 min or treated with 100 μg/ml Ot-WHP for different time points (0, 5, 15, 30, 60 min). For Smad signaling assay, BMDMs were incubated with Ot-WHP (0, 25, 50, and 100 μg/ml) for 24 h, or incubated with Ot-WHP (100 μg/ml) for different time points (0, 6, 12, and 24 h). Considering that Smad2/3 signal pathways are dependent on TGF-β signal (39), the effect of Ot-WHP (100 μg/ml) on Smad signal pathways in BMDMs were also assayed in the presence or absence anti-TGF-β1 antibody (10 μg/ml, abcam, USA). BMDMs were then collected, centrifuged at 1000 × *g* for 5 min, washed twice with ice-cold PBS, and lysed with RIPA lysis buffer (Beyotime, China). About 40 μg total protein was separated on a reducing SDS-PAGE gel (12%) and transferred onto a polyvinylidene difluoride (PVDF) membrane. The immunoblot was blocked with 5% BSA (BD Biosciences) at room temperature for 3 h, followed by an overnight incubation with a primary antibody against ERK, phosphorylated ERK, JNK, phosphorylated JNK, NF-κB p65, phosphorylated NF-κB p65, phosphorylated IκBα, Smad2, phosphorylated Smad2, Smad3, phosphorylated Smad3 and β-actin (1:2,000, Cell Signaling Technology, Massachusetts, USA) at 4°C. The signals were measured with secondary antibody (1:5,000, Cell Signaling Technology, Massachusetts, USA) for 1 h at room temperature using an enhanced chemiluminescence kit (Tiangen Biotech, Beijing, China).

### Neutrophil and Macrophage Migration Assay

Neutrophils or BMDMs (7 × 10^6^ cells/ml, 100 μl), in RPMI 1640 supplemented with 2% FBS, were added to the 3.0-μm-pore-size Transwell filters (the upper chamber) in a 24-well format, and 500 μl of Ot-WHP (25, 50, 100 μg/ml, dissolved in 2% FBS RPMI 1640 medium) or medium, was placed in the lower chamber. After being cultured for 8 h at 37 °C, cells in the lower chamber were collected and counted using a hemocytometer. The increased cells in the lower chamber were the migrated cells.

For co-cultured system, BMDMs (4 × 10^6^ cells/ml, 500 μl) were seeded to the lower chamber in RPMI 1640 supplemented with 2% FBS. After BMDMs in the lower chamber were adhered to the plate, neutrophils or BMDMs (7 × 10^6^ cells/ml, 100 μl) were added to the 3.0 μm-pore-size Transwell filters (the upper chamber) in a 24-well format, and 500 μl of Ot-WHP (25, 50, 100 μg/ml, dissolved in 2% FBS RPMI 1640 medium) or medium, was added to BMDMs in the lower chamber. After being cultured for 8 h at 37°C, cells in the upper chamber were collected and counted using a hemocytometer. The reduced cells in the upper chamber were the migrated cells (40).

### Wound Healing Scratched Assay

HaCaT cells (1 × 10^6^/ml) were seeded into a 6-well plate and grown to monolayer confluency. After serum starvation (DMEM supplemented with 1% FBS) for 24 h, the cell monolayer was scratched with a sterile pipette tip to create a wound slit about 1 mm width. After washing twice with PBS to remove floating cells, cells were then cultured for additional 48 h in a serum-free basal medium in the presence of Ot-WHP (25, 50, 100 μg/ml), a same volume of vehicle (PBS, negative control), or AH90 (25, 50, 100 μg/ml, positive control). Mitomycin C (10 μg/ml) was always included in the media to prevent cell proliferation. Images of the wounded cell monolayers at 0, 24, and 48 h after scratch wounding were obtained using a microscope (Olympus, Japan). Image J software (U.S. National Institutes of Health, Bethesda, MD, USA) was used to calculate the repair rate of scarification, which represented cell migration activity and was expressed as the percentage of the gap relative to the total area of the cell-free region immediately after scratch wounding (24).

### Flow Cytometry Assay

For assaying the integrin subunits on surface of keratinocytes, HaCaT cells (5 × 10^5^ cells/well, 6-well plate) were incubated with Ot-WHP (100 μg/ml) or 2% BSA in PBS for 30 min, and washed three times with PBS. The cells were incubated with PE-conjugated anti-β1 (CD29, cat number, 303003) or isotype control (mouse IgG1, cat number, 400111), anti-α3 (CD49c, cat number, 343803) or isotype control (mouse IgG1, cat number, 400111), anti-α5 (CD49e, cat number, 328009) or isotype control (Mouse IgG2b, cat number, 400311), anti-α6 (CD49f, cat number, 313611) isotype control (Rat IgG2a, cat number, 400507) antibodies (BioLegend) for 30 min at 4°C. After incubation, HaCaT cells were washed with PBS three times (24).

All the prepared cells were re-suspended in PBS, assayed on a FACScalibur flow cytometer, and analyzed by Cell Quest software (BD Immunocytometry).

### Cell Adhesion Assay

Fibronectin (20 μg/ml), laminin (20 μg/ml), or BSA (2%, control) were dissolved in PBS and pre-coated on 96-well plates. After treatment with Ot-WHP (25, 50, 100 μg/ml) or vehicle (PBS) overnight, HaCaT cells were seeded in the pre-coated plates at a density of 5 × 10^3^ cells per well. After incubation at 37°C for 60 min, the cells were washed with PBS, and the adherent cells were left. Then the cells were fixed in 4% paraformaldehyde and washed twice with PBS, stained with crystal violet for 10 min, washed twice with PBS, and lysed with 2% SDS. Absorbance at 490 nm was measured on a microplate reader (BioTek, Vermont, USA). Background values (binding to BSA) were subtracted, and the number of adherent cells with vehicle treatment was arbitrary determined as 100% adhesion (24).

### Cell Proliferation Assay

Cells were seeded (5 × 10^3^ cells/well, 100 μl/well) in 96-well plates, and incubated with Ot-WHP (25, 50, and 100 μg/ml) or the same volume of vehicle (PBS) for 24 h (BMDMs, THP-1, keratinocytes) or 72 h (fibroblasts), respectively. Then cell counting kit-8 (CCK-8) solution (10 μl) was added to each well to incubate at 37°C for further 2–4 h. The absorbance at 450 nm was measured on a microplate reader (BioTek, Vermont, USA).

### Co-culture of Macrophages With Keratinocytes/Fibroblasts

Keratinocytes or fibroblasts were co-cultured with macrophages as described previously (33). Human keratinocytes (HaCaT, 1 × 10^5^ cells/well) were co-cultured with THP-1-derived macrophages (1 × 10^5^ cells/well), and mouse skin-derived fibroblasts (1 × 10^5^ cells/well) were co-cultured with BMDMs (1 × 10^5^ cells/well), respectively. Keratinocytes or fibroblasts were seeded in the lower chamber (24-well plates). After culture for 24 h at 37°C, keratinocytes/fibroblasts were adhered to the culture plates. Macrophages or the same volume of medium (100 μl) were added to the upper chamber of the transwell inserts. Ot-WHP (25, 50, 100 μg/ml) or a same volume of vehicle (PBS) was added to the upper chamber. Keratinocytes and fibroblasts were co-cultured with macrophages for 24 and 72 h, respectively. After co-culture, macrophages in the upper chambers were discarded, and the cell proliferation of keratinocytes/fibroblasts in the lower chamber was assayed using CCK-8 kit, the accumulation of collagen in the medium of fibroblasts was quantified by an ELISA kit (Shanghai Yuanye Bio-Technology, China), and the expression of α-SMA in fibroblasts was detected by Western blot as mentioned above. A primary anti-α-SMA (1:2,000, Cell Signaling Technology, Massachusetts, USA) antibody was used for Western blot analysis.

### Statistical Analysis

Statistical analysis was performed using Student's *t-*tests or one- way ANOVA provided by GraphPad Prism software (GraphPad Software Inc., La Jolla, CA, USA). Data were presented as mean ± standard deviation from three independent experiments. *p* < 0.05 was considered as a statistically significant difference between the groups.

## Results

### A Wound Healing-Promoting Peptide, Ot-WHP, Was Isolated From the Skin of *O. tormotus*

**Figure 1:**
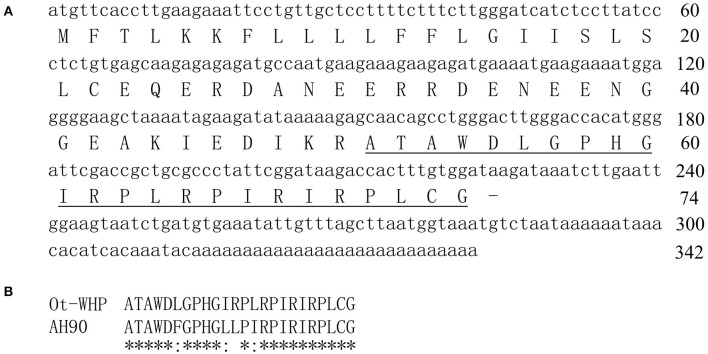
The nucleotide sequence encoding Ot-WHP precursor **(A)**, the deduced amino acid sequence **(A)** and alignment of Ot-WHP with AH90 **(B)**. The sequence of mature peptide is underline, and line segment (-) indicates stop codon. The identical sites (*) and conserved sites (:) were indicated in the multiple alignment of Ot-WHP with an *Odorrana graham*-derived wound healing-promoting peptide (AH90).

To isolate *O. tormotus*-derived wound healing-promoting peptides, the skin secretions were first separated by Sephadex G-50 gel filtration as shown in Figure S1A. The fraction containing wound healing-promoting activity in cutaneous wounds of mice (indicated by an arrow) was subjected to a C18 RP-HPLC column twice over (Figures S1B,C). The purified wound healing-promoting peptide (named Ot-WHP) was subjected to mass spectrometry (MS) analysis and had an observed molecular weight of 2666.12 Da, which matched well with the theoretical molecular weight of 2666.19 Da. The amino acid sequence of Ot-WHP was composed of 24 amino acid residues and determined as ATAWDLGPHGIRPLRPIRIRPLCG by Edman degradation. The complete nucleotide sequence (GenBank accession number, MK431780) that encoded the precursor of Ot-WHP was cloned from the skin cDNA library and contained 342 base pairs (Figure 1A). The precursor of Ot-WHP was composed of 74 amino acid residues (Figure 1A). The amino acid sequence of the deduced mature Ot-WHP is consistent with that obtained by Edman degradation (Figure 1). Blast search indicated that the naturally occurring Ot-WHP is a small peptide with different amino acid sequences as compared to other amphibian-derived wound healing-promoting peptides described previously. In addition, Ot-WHP shows 83.3% sequence identity with an *Odorrana graham*-derived wound healing-promoting peptide (AH90, Figure 1B) (24), implying that Ot-WHP probably has wound-healing promoting potential.

### Ot-WHP Efficiently Accelerated the Healing of Full-Thickness Wounds in Mice

**Figure 2:**
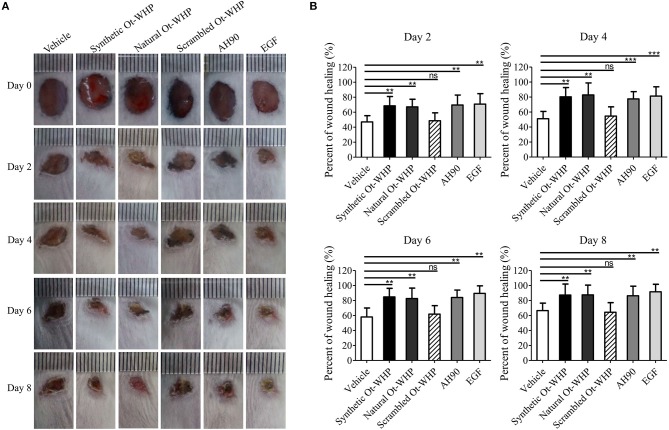
Topical application of Ot-WHP accelerated the healing of full-thickness wounds in mice. **(A)** Photos of wounds. **(B)** Wound healing rates. Peptide or EGF samples were dissolved in PBS. Each wound was treated with vehicle (20 μl, PBS), synthetic Ot-WHP (20 μl, 200 μg/ml), natural Ot-WHP (20 μl, 200 μg/ml), scrambled Ot-WHP (20 μl, 200 μg/ml), AH90 (20 μl, 200 μg/ml), or EGF (20 μl, 100 μg/ml, epidermal growth factor) one time per day from days 0 to 8, respectively. Wound area was monitored by taking digital photographs at days 0, 2, 4, 6, and 8, and the wound area was calculated from the photographs using PhotoShop (Adobe Photoshop Element 2.0, Adobe Systems, San Jose, CA, USA). The wound healing rate of each wound at days 2, 4, 6, and 8 was calculated form the wound area of day 2/day 0 × 100%, day 4/day 0 × 100%, day 6/day 0 × 100%, and day 8/day 0 × 100%, respectively. ns, no significance, **p < 0.01, ***p < 0.001.

A mouse model of full-thickness wounds was used to identify and evaluate Ot-WHP *in vivo*. As shown in Figure 2, Ot-WHP treatment (both synthetic Ot-WHP and natural Ot-WHP, 20 μl/wound/day, 200 μg/ml) significantly accelerated the full-thickness wound repair in mice as compared to vehicle (PBS) treatment. At days 2, 4, 6, and 8, the wound healing rates observed in synthetic Ot-WHP treated mice were 67.7, 81.5, 84.8, and 88.5%, and the wound healing rates observed in natural Ot-WHP-treated mice were 67.2, 79.7, 85.8, and 87.5%, respectively. Both synthetic Ot-WHP and natural Ot-WHP showed comparable wound healing-promoting capacity to AH90 (a positive control, peptide) (24) and EGF (a positive control, epidermal growth factor) in a mouse model of dermal full-thickness wounds. Whereas, scrambled Ot-WHP (an isotype control, peptide) did not show any wound healing-promoting effect, which indicated the specific effect of Ot-WHP on wound healing. In addition, no adverse effects on the body health or behavior of the mice were found in any group after the topical treatment.

**Figure 3:**
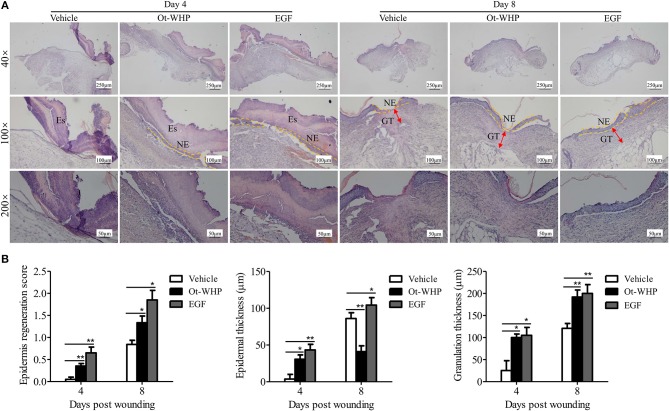
Histopathological study of the effects of Ot-WHP on the healing of full-thickness wounds in mice. **(A)** Each wound in BABL/c mice (*n* = 6) was treated with vehicle (PBS, 20 μl/wound/day), Ot-WHP (20 μl/wound/day, 200 μg/ml), or EGF (20 μl, 100 μg/ml, positive control), and wounds were taken at days 4 and 8 post injury and stained with H&E (Es, eschar; GT, granulation tissue; NE, neo-epithelium). **(B)** Histological scores of epidermis regeneration, epidermal thickness and granulation thickness of the wound tissue sections. *p < 0.05, **p < 0.01.

Histopathological study showed that Ot-WHP treatment improved the epidermal regeneration, promoted the granulation tissue formation and formed thinner epidermal layers in mice, as compared to vehicle treatment (Figure 3). At day 4 post injury, vehicle (PBS)-treated wounds had severe edema with many swelling endothelial cells, while Ot-WHP-treated wounds exhibited fragmentary epidermal tissues and well-formed granulation tissues. At day 8 post injury, Ot-WHP-treated wounds showed a more complete epidermal regeneration and thicker granulation tissue to vehicle-treated group. Additionally, the thinner epithelium observed in Ot-WHP-treated group indicated that no epithelial hyperproliferation was induced by Ot-WHP treatment.

### Ot-WHP Enhanced the Recruitment of Neutrophils to Wound Sites in Mice

**Figure 4:**
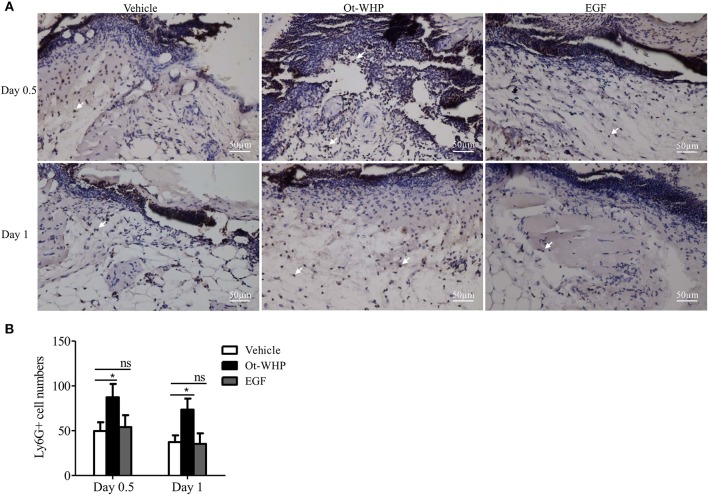
Ot-WHP increased the numbers of neutrophils in wounds. **(A)** Images of wound sections stained with anti-mouse Ly6G. **(B)** Number of neutrophils per microscopic field. Each wound in BABL/c mice (*n* = 6) was treated with vehicle (PBS, 20 μl/wound/day), Ot-WHP (20 μl/wound/day, 200 μg/ml), or EGF (20 μl, 100 μg/ml, positive control), and wounds were taken at days 0.5 and 1 post injury. The biopsy of wounds including healed tissues (about 8 mm in diameter) were embedded in paraffin wax, sectioned and incubated with rat anti-mouse Ly6G antibody, number of neutrophils (Ly6G+ cells, indicated by arrows) per microscopic field in a 40 × objective were calculated by Image J from three random fields of each wound from 6 mice. ns, no significance, *p < 0.05.

Neutrophils arrive first within a few minutes in the inflammatory phase after injury, which constitutes the first step in wound healing and is essential for efficient tissue repair (41, 42). To see if amphibian-derived wound healing peptide acts on neutrophils during wound healing, we first investigated the effect of Ot-WHP on neutrophil infiltration in the wound sites of mice. As shown in Figure 4, Ot-WHP treatment significantly enhanced neutrophil infiltration in the wound sites as compared to vehicle (PBS) treatment. At days 0.5 and 1, neutrophil infiltration in Ot-WHP-treated wounds increased by 95.5 and 84.1%, respectively. The data indicated that Ot-WHP efficiently enhanced the recruitment of neutrophils to the wound sites of mice.

### Ot-WHP Modestly Promoted Neutrophil Phagocytosis and PMA-Induced Neutrophil Extracellular Trap Formation

**Figure 5:**
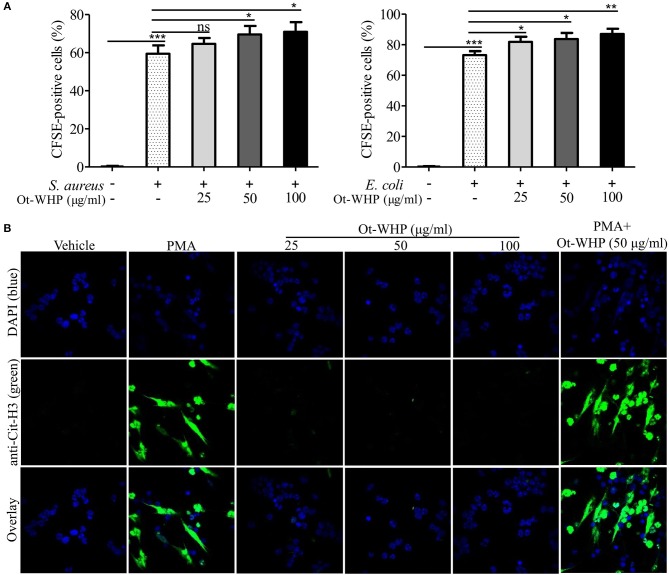
Ot-WHP modestly promoted neutrophil phagocytosis and PMA-induced neutrophil extracellular trap formation. **(A)** Ot-WHP (25, 50, 100 μg/ml) modestly promoted neutrophil phagocytosis against *S. aureus* and *E. coli*. Neutrophils were pre-incubated with peptide or vehicle (PBS) for 1 h, and CFSE-labeled bacterial particles were added and incubated for 1.5 h. **(B)** Effect of Ot-WHP on NET formation. Neutrophils were incubated with vehicle (PBS), Ot-WHP (25, 50, 100 μg/ml), PMA (100 nM), or PMA (100 nM) + Ot-WHP (50 μg/ml) at 37°C for 4 h, respectively. Nuclei and NETs were stained with DAPI (blue) or anti-Cit-H3 (green), respectively. NETs were observed using a confocal microscope (×60, Nikon, Japan). The average of 3 independent experiments is shown. ns, no significance, *p < 0.05, **p < 0.01, ***p < 0.001.

The infiltrated neutrophils initiate debridement of cell debris and pathogens in the wounds (41, 42). We hence detected the effect of Ot-WHP on neutrophil phagocytosis and NET formation. As shown in Figure 5A, Ot-WHP modestly promoted *in vitro* phagocytosis of fluorescently labeled *S. aureus* and *E. coli* by neutrophils in a dose dependent manner. Neutrophil extracellular traps (NETs) are extremely critical for clearing off cell debris and microbes in wounds (42). We next investigated the effect of Ot-WHP on NET formation. As shown in Figure 5B, PMA (100 nM, positive control) treatment markedly induced NET formation in neutrophils, while Ot-WHP treatment had no significant effects on NETs formation at a concentration up to 100 μg/ml. However, Ot-WHP (50 μg/ml) could modestly promote PMA-induced NET formation. The data implied that Ot-WHP possibly enhanced NET formation upon cell debris or pathogen stimulation, despite having no direct effect on NET formation. The infiltrated neutrophils also secreted pro-inflammatory cytokines that serve to activate local fibroblasts and keratinocytes (41). We also investigated whether Ot-WHP induced cytokine as well as chemokine production in neutrophils. As shown in Figure S2, Ot-WHP did not induce the production of any of the tested cytokines nor chemokines by neutrophils at a concentration up to 100 μg/ml.

### Ot-WHP Promoted the Recruitment of Macrophages to Wounds

**Figure 6:**
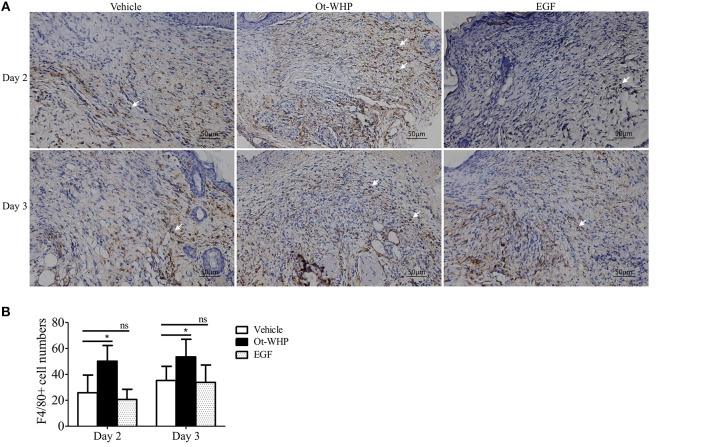
Ot-WHP increased the numbers of macrophages in wounds. **(A)** Images of wound sections stained with anti-mouse F4/80. **(B)** Number of macrophages per microscopic field. Each wound in BABL/c mice (*n* = 6) was treated with vehicle (PBS, 20 μl/wound/day), Ot-WHP (20 μl/wound/day, 200 μg/ml) or EGF (20 μl, 100 μg/ml, positive control), and wounds were taken at days 2 and 3 post injury. The biopsy of wounds including healed tissues (about 8 mm in diameter) were embedded in paraffin wax, sectioned and incubated with rat anti-mouse F4/80 antibody, number of macrophages (F4/80+ cells, indicated by arrows) per microscopic field in a 40 × objective were calculated by Image J from three random fields of each wound from 6 mice. ns, no significance, *p < 0.05.

At the inflammatory phase of wound healing, macrophages constitute another important infiltrated immune cell type after neutrophil infiltration (42). We were then interested to investigate whether Ot-WHP affected macrophage infiltration. As shown in Figure 6, a large number of macrophages were recruited to wound sites at days 2 and 3 post injury, and Ot-WHP treatment significantly enhanced the recruitment of macrophages to the wound sites as compared to vehicle (PBS) treatment. At days 2 and 3, macrophages recruited to Ot-WHP-treated wounds were increased by 94.6 and 99.1%, respectively. This suggested that Ot-WHP efficiently enhanced the recruitment of macrophages to wound sites.

### Ot-WHP Directly Induced the Production of Chemokines, Cytokines and Growth Factor in BMDMs and Wound Sites

**Figure 7:**
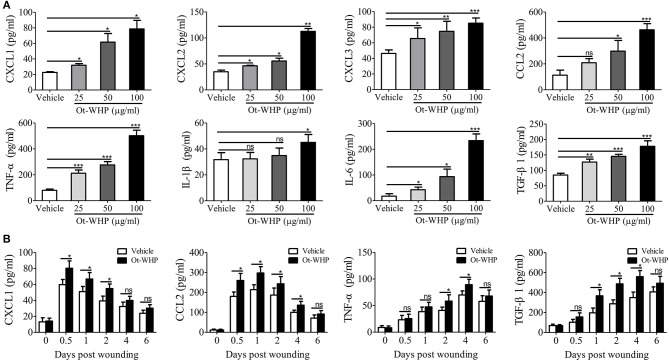
Ot-WHP induced chemokine and cytokine production in mouse BMDMs and wound sites. **(A)** After treatment for 24 h, Ot-WHP (25, 50, 100 μg/ml) induced chemokine (CXCL1, CXCL2, CXCL3, and CCL2) and cytokine (TNF-α, IL-1β, IL-6, and TGF-β1) production in BMDMs in a dose-dependent manner. **(B)** Ot-WHP regulated chemokine (CXCL1 and CCL2) and cytokine (TNF-α) and transforming growth factor (TGF-β1) production in wound sites at indicated time points post injury. A same volume of vehicle (PBS) served as control. The biopsy of wound including healed tissues (about 8 mm in diameter) from each mouse (*n* = 6) was taken at days 0, 0.5, 1, 2, 4, and 6, and was ground into homogenate in 0.1 M PBS (containing 1 mM PMSF, 1 ml/g tissue) by a glass homogenizer. The levels of cytokine and chemokine in the culture medium of BMDMs and the supernatant of wound homogenates were quantified by ELISA. The average of 3 independent experiments is shown. ns, no significance, *p < 0.05, **p < 0.01, ***p < 0.001.

To understand how Ot-WHP enhanced the recruitment of neutrophils and macrophages to wound sites, we tested whether Ot-WHP could induce the production of chemokines and cytokines in mouse bone marrow-derived macrophages (BMDMs). As shown in Figure 7A, Ot-WHP significantly induced the chemokine and cytokine production in mouse BMDMs in a dose-dependent manner. At a concentration of 100 μg/ml, CXCL1, CXCL2, CXCL3, CCL2, TNF-α, IL-1β, and IL-6 levels in the supernatant of Ot-WHP-treated BMDMs were increased by 2.5, 2.2, 0.8, 3.1, 5.3, 0.4, and 12.2-folds as compared to vehicle (PBS)-treated BMDMs. A similar result was observed *in vivo*, Ot-WHP treatment also significantly enhanced the chemokine and cytokine production in wound sites relative to vehicle (PBS) treatment (Figure 7B). Ot-WHP treatment significantly enhanced CXCL1, CCL2 production in wound sites at days 0.5, 1, 2, and 4 post injury, and significantly enhanced TNF-α production in wound sites at days 2 and 4 post injury. The data suggested that Ot-WHP directly activated macrophages and significantly enhanced the production of chemokines and cytokines both *in vitro* and *in vivo*, which in turn efficiently promoted the recruitment of neutrophils and macrophages to wound sites. In addition, Ot-WHP treatment also significantly up-regulated the production of TGF-β1 in BMDMs and wound sites (Figure 7), which is a critical growth factor for the healing of acute cutaneous wounds. TGF-β1 production level in BMDMs was increased by 1.1-fold following Ot-WHP (100 μg/ml) treatment relative to vehicle (PBS) treatment (*p* < 0.001), and TGF-β1 production levels in wound sites were increased by 0.9, 0.7, and 0.6-fold at days 1, 2, and 4 post injury, following Ot-WHP treatment relative to vehicle (PBS) treatment, respectively (*p* < 0.5).

### Ot-WHP Did Not Act as a Chemoattractant for Neutrophils and Macrophages

**Figure 8:**
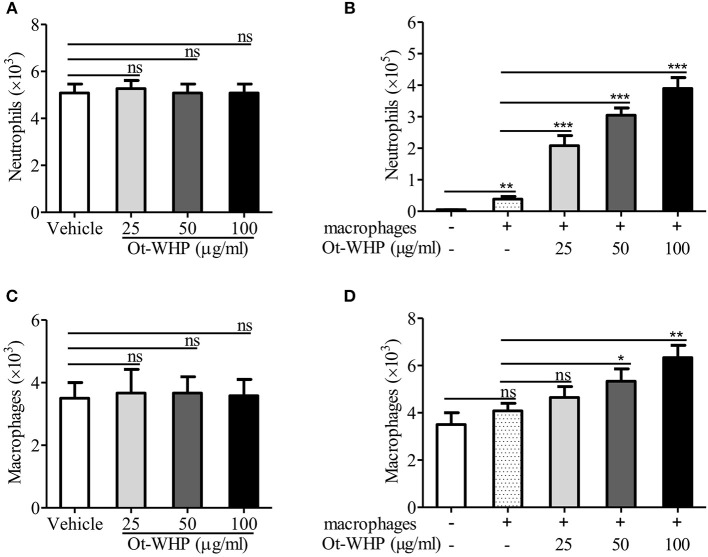
Ot-WHP did not act as a chemoattractant for neutrophils **(A)** or BMDMs **(C)** alone, but it was chemotactic to neutrophils **(B)**, and BMDMs **(D)** in the presence of BMDMs. **(A,C)** Neutrophil or BMDM suspension (7 × 10^6^ cells/ml, 100 μl) was added to the upper chamber. 500 μl of Ot-WHP or vehicle (medium) was placed in the lower chamber. Neutrophils and macrophages were migrated at 37 °C for 8 h. Then, cells in the lower chamber were collected and counted using a hemocytometer. **(B,D)** Mouse BMDMs (4 × 10^6^ cells/ml, 500 μl) were seeded and adhered to the lower chamber. Neutrophils or BMDMs (7 × 10^6^ cells/ml, 100 μl) were added to the 3.0 μm-pore-size Transwell filters (upper chamber) in a 24-well format. Then, 500 μl of Ot-WHP or vehicle (medium) was added to BMDMs in the lower chamber. Neutrophils and macrophages were migrated at 37°C for 8 h. Then, cells in the upper chamber were collected and counted using a hemocytometer. The average of 3 independent experiments is shown. ns, no significance. *p < 0.05, **p < 0.01, ***p < 0.001.

Given the increase in neutrophils and macrophages at the wound site, we were interested to test whether Ot-WHP could directly induce neutrophil and macrophage migration. As shown in Figures 8A,B, Ot-WHP had no direct effect on neutrophil and macrophage migration. It suggested that Ot-WHP did not act as a chemoattractant for neutrophils and macrophages. As observed above, Ot-WHP directly induced the production of chemokines as well as cytokines in macrophages. We next tested the effect of Ot-WHP on neutrophil and macrophage migration in the presence macrophages. As shown in Figures 8C,D, Ot-WHP significantly enhanced neutrophil and macrophage migration in a dose-dependent manner in the presence of macrophages. At a concentration of 100 μg/ml, Ot-WHP induced about 3.51 × 10^5^ neutrophil migration and 2.25 × 10^3^ macrophage migration in the co-cultured system. In combination with the chemokine and cytokine-inducing capacity of the peptide, we can conclude that Ot-WHP enhanced neutrophil and macrophage migration via inducing chemoattractant production in macrophages.

### Ot-WHP Directly Activated MAPKs and NF-κB Signaling Pathways in BMDMs

**Figure 9:**
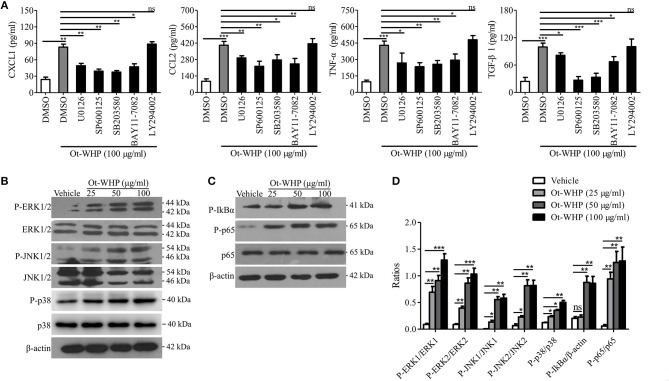
Ot-WHP exerted its immunoregulatory effects on BMDMs via activating MAPKs and NF-κB signaling pathways. **(A)** Effects of chemical inhibitors against MAPKs, NF-κB, and PI3K on Ot-WHP-induced chemokine (CXCL1 and CCL2) and cytokine (TNF-α) and transforming growth factor (TGF-β1) production in mouse BMDMs. BMDMs were pre-incubated with ERK inhibitor (U0126, 10 μM), JNK inhibitor (SP600125, 10 μM), p38 inhibitor (SB203580, 10 μM), NF-κB inhibitor (BAY11-7082, 2 μM), or PI3K inhibitor (LY294002, 10 μM) for 1 h, respectively, and then were stimulated with Ot-WHP (100 μg/ml) for 24 h. The levels of regulatory factors in BMDMs were quantified by ELISA. **(B–D)** Ot-WHP (25, 50, 100 μg/ml) markedly activated MAPKs (ERK and JNK subgroups) **(B)** and NF-κB (IκBα and p65 subgroups) **(C)** signaling pathways in mouse BMDMs, and the phosopholyation of ERK, JNK, IκBα, and p65 were quantified by Image J **(D)**. A same volume of vehicle (PBS) was used as control. The average of 3 independent experiments is shown. ns, no significance, *p < 0.05, **p < 0.01, ***p < 0.001.

To investigate the downstream signaling pathways involved in the immunomodulatory activity of the peptide, chemical inhibitor of MAPKs, NF-κB, or PI3K was co-cultured with Ot-WHP in BMDMs, respectively, and the production levels of CXCL1, CCL2, TNF-α, and TGF-β1 were tested. As shown in Figure 9A, the addition of chemical inhibitor of MAPKs or NF-κB significantly blocked the production of CXCL1, CCL2, TNF-α, and TGF-β1 in Ot-WHP-stimulated BMDMs. In contrast, inhibition of PI3K did not have a significant effect on Ot-WHP activity. Activation of MAPK and NF-κB signaling pathways by Ot-WHP were further confirmed by western blot (Figures 9B–D). Ot-WHP significantly induced the activation (phosphorylation) of MAPKs (ERK, JNK and p38 subgroups) and NF-κB (IκBα and p65 subgroups) in a dose-dependent manner. For instance, the phosphorylation levels of ERK1, ERK2, JNK1, JNK2, p38, IκBα, and p65 were increased by 9.3, 8.8, 91.5, 11.1, 1.9, 3.3, and 20.7 folds after treatment of 50 μg/ml Ot-WHP relative to vehicle (PBS) treatment, respectively. The data suggested that MAPKs (ERK and JNK subgroups) and NF-κB (IκBα and p65 subgroups) were involved in Ot-WHP-induced chemokine, cytokine and growth factor production in BMDMs.

### Ot-WHP Activated TGF-β-Dependent Smad Signaling Pathway in BMDMs

**Figure 10:**
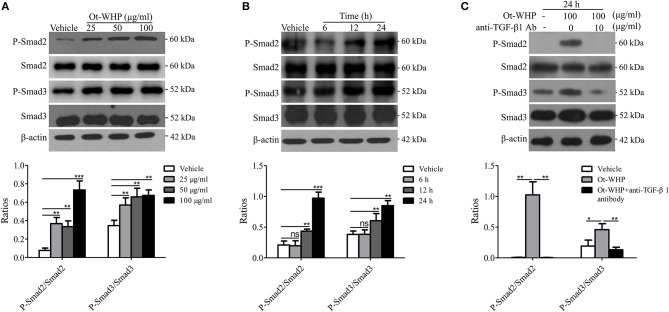
Ot-WHP activated TGF-β-dependent Smad signaling pathway in BMDMs. **(A)** Ot-WHP (25, 50, 100 μg/ml) significantly induced the phosphorylation of Smad2 and Smad3 in mouse BMDMs in a dose-dependent manner. **(B)** Time-course assay of the effect of Ot-WHP (100 μg/ml) on the phosphorylation of Smad2 and Smad3 in mouse BMDMs. **(C)** Ot-WHP (100 μg/ml) induced the phosphorylation of Smad2 and Smad3 in mouse BMDMs in a TGF-β-dependent manner. Anti-mouse TGF-β1 antibody (10 μg/ml) was used to neutralize transforming growth factor (TGF-β1) in BMDMs induced by Ot-WHP. The ratios were quantified by Image J. A same volume of vehicle (PBS) was used as control. The average of 3 independent experiments is shown. ns, no significance, *p < 0.05, **p < 0.01, ***p < 0.001.

Smad family proteins, including Smad2 and Smad3, are essential components of downstream TGF-β signaling, and key modulators of TGF-β signaling (39, 43). As mentioned above, Ot-WHP significantly elicited the production of TGF-β1 in BMDMs. We next detected whether Ot-WHP activated the downstream of TGF-β signaling. As shown in Figure 10A, Ot-WHP significantly induced the activation (phosphorylation) of Smad2 and Smad3 in a dose-dependent manner in BMDMs after incubation for 24 h. The time-course of Ot-WHP-induced Smad signaling activation was also assayed. As illustrated in Figure 10B, Ot-WHP (100 μg/ml) did not induce the activation of Smad signaling pathway in BMDMs at 6 h after the addition of Ot-WHP (100 μg/ml), while the activation of Smad signaling pathway in BMDMs appeared at 12 h and was further enhanced at 24 h after the addition of Ot-WHP (100 μg/ml). Ot-WHP could not cause a rapid activation of Smad signaling pathway, which implied that Ot-WHP-induced Smad signaling activation in BMDMs might be dependent on TGF-β1 secretion. In order to prove this speculation, TGF-β1 antibody (10 μg/ml) was used to neutralize TGF-β1 in Ot-WHP-stimulated (100 μg/ml) BMDMs. After the addition of TGF-β1 antibody (10 μg/ml), Ot-WHP-induced Smad signaling activation in BMDMs was completely blocked even incubation for 24 h (Figure 10C). This suggested that Ot-WHP markedly activated the downstream TGF-β signaling, Smad2 and Smad3, in BMDMs via inducing TGF-β1 production.

### Ot-WHP Directly Enhanced the Migration of Keratinocytes by Promoting Integrin Expression and Cell Adhesion

**Figure 11:**
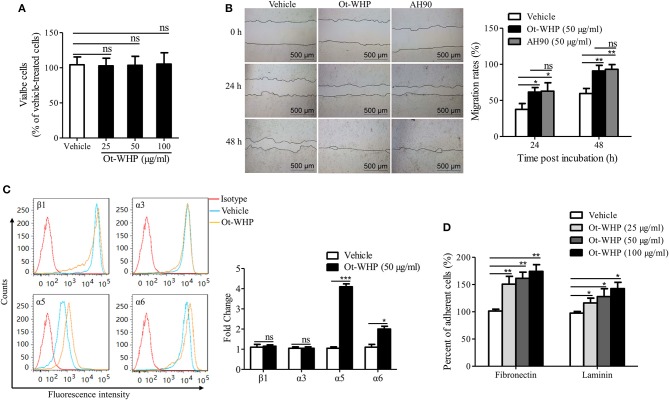
Ot-WHP enhanced the migration of keratinocytes by promoting cell adhesion and integrin expression. **(A)** Ot-WHP (25, 50, 100 μg/ml) had no significant effect on keratinocyte (HaCaT) proliferation. **(B)** Ot-WHP (50 μg/ml) enhanced the migration of keratinocytes (HaCaT). AH90 (100 μg/ml) served as positive control. **(C)** Effect of Ot-WHP (50 μg/ml) on the expression of integrin β1, α3, α5, and α6 subunit on cell keratinocyte (HaCaT) surface. The expression levels of integrin subunits were assayed by flow cytometry, and quantified by fold change. **(D)** Ot-WHP (25, 50, 100 μg/ml) promoted keratinocyte (HaCaT) cell adhesion to laminin (20 μg/ml) or fibronectin (20 μg/ml). A same volume of vehicle (PBS) served as control for each experiment. The average of 3 independent experiments is shown. ns, no significance, *p < 0.05, **p < 0.01, ***p < 0.001.

The data above suggested neutrophils and macrophages constituted two important effector cell types of Ot-WHP. In addition, keratinocytes and fibroblasts are also critical cell types in wound healing. Herein, we were interested in whether keratinocytes and fibroblasts were effector cells of Ot-WHP in wound healing. We first detected the direct effect of Ot-WHP on HaCaT cell proliferation. As illustrated in Figure 11A, Ot-WHP did not have any effect on HaCaT cell proliferation at a concentration up to 100 μg/ml. We next detected its effect on keratinocyte cell migration, which is the most critical factor in promoting re-epithelialization and accelerating the wound closure (24). An *in vitro* cell scratch assay indicated that Ot-WHP (50 μg/ml) significantly promoted keratinocyte migration as compared to vehicle (PBS)-treated keratinocytes (Figure 11B). At 24 and 48 h post scratch, the HaCaT cell migration rates of the scarification induced by Ot-WHP were 61.6 and 90.8%, while those induced by vehicle (PBS) were just 37.5 and 59.6%, and the migration rates induced by Ot-WHP were comparable to those induced by AH90 (a positive control, peptide). The data suggested that Ot-WHP acted directly on keratinocytes by promoting keratinocyte migration.

Integrins are heterodimeric transmembrane proteins consisting of an α and a β subunit that play crucial roles in cell-cell and cell-matrix interaction, which regulate cell adhesion and cell spreading, as well as migration, proliferation, differentiation and remodeling of the extracellular matrix (43). The expression profile of integrins on wound margin of keratinocytes changes after wounding, which is characterized by suprabasal integrin expression and induction of specific integrins (24). In order to understand the mechanism of HaCaT cell migration induced by Ot-WHP, the effects of Ot-WHP (50 μg/ml) on expression of integrins (including β1, α3, α5, and α6) at the surface of keratinocytes were assayed. As illustrated in Figure 11C, the expressions of α5 and α6 integrins on the cell surface were up-regulated in Ot-WHP-treated keratinocytes, whereas those of β1 and α3 subunits had no significant changes. The ligands of integrins that contain α5 or α6 are α5β1, α6β1, and α6β4, of which α5β1 is fibronectin, α6β1, and α6β4 are laminin (43, 44). Fibronectin and laminin primarily act as cell-matrix interaction (45). Since Ot-WHP up-regulated the expression of α5 and α6 integrins, adhesion assays on fibronectin and laminin were investigated. As a result, adhesions of Ot-WHP-treated keratinocytes to fibronectin and laminin were significantly up-regulated (Figure 11D). At concentrations of 25, 50, and 100 μg/ml, the cell adhesion to fibronectin was increased by 50.8, 61.4, and 74.3%, and the cell adhesion to laminin was increased by 16.3, 27.9, and 42.6% following Ot-WHP treatment, respectively. The result confirmed the capacity of Ot-WHP to up-regulate the expressions of α5 or α6 subunit. These results indicated that Ot-WHP directly enhanced the migration of keratinocytes by promoting integrin expression and cell adhesion.

### Ot-WHP Indirectly Induced Fibroblast-to-Myofibroblast Transition and Collagen Production in Mouse Wound Sites

**Figure 12:**
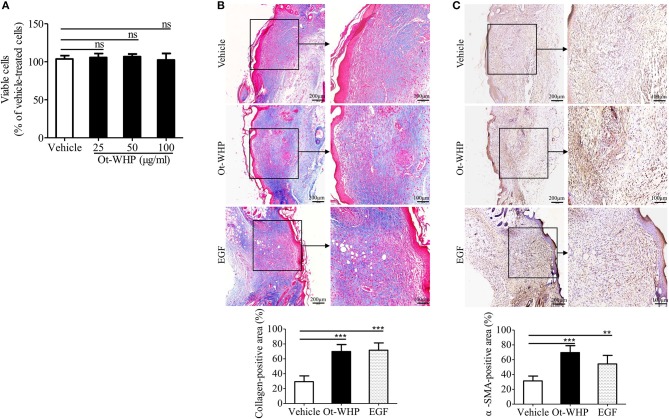
Ot-WHP significantly promoted collagen deposition and fibroblast-to-myofibroblast transition in wounds. **(A)** Ot-WHP (25, 50, 100 μg/ml) had no significant effect on the proliferation of fibroblasts (isolated from newborn mouse skin). **(B)** Histological assessment of collagen deposition in wound sites. **(C)** Histological analysis of the expression of α-SMA in wound sites by immunohistochemistry. Each wound in BABL/c mice (*n* = 6) was treated with vehicle (PBS, 20 μl/wound/day), Ot-WHP (20 μl/wound/day, 200 μg/ml) or EGF (20 μl, 100 μg/ml, positive control), and biopsy of wounds including healed tissues (about 8 mm in diameter) were taken at day 8 post injury. After the wound tissues were embedded in paraffin wax and sectioned into 5 μm slices, the slices were stained with Masson Trichrome for collagen deposition assay, or incubated with rat anti-mouse α-SMA antibody. Blue-stained (collagen-positive) area or brown-stained (α-SMA-positive) area from each section was determined using color-based thresholding by Image J. Three microscopic fields in a 10 × objective of each section were randomly selected, and percent of positive area was calculated from the wound sections from 6 mice. The average of 3 independent experiments is shown. ns, no significance, **p < 0.01, ***p < 0.001.

**Figure 13:**
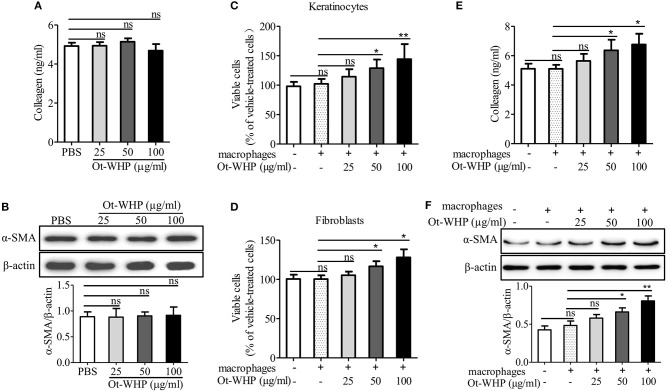
Ot-WHP had no significant effect on collagen production **(A)** and α-SMA expression **(B)** in fibroblasts without co-culture of macrophages, but it enhanced keratinocyte **(C)** and fibroblast **(D)** proliferation, and collagen production **(E)** and α-SMA expression **(F)** in fibroblasts in co-culture with macrophages. **(A,B)** Fibroblasts were isolated from newborn mice. After the addition of Ot-WHP (25, 50, 100 μg/ml) or vehicle (PBS) for 72 h, collagen accumulation in the culture medium of fibroblasts **(A)** and α-SMA expression in fibroblasts **(B)** were determined by ELISA and Western blot, respectively. **(C–F)** Keratinocytes (HaCaT, in the lower chamber) were co-cultured with THP-1 derived macrophages (in the upper chamber) for 24 h. Fibroblasts (in the lower chamber) isolated from newborn mice skin were co-cultured with mouse BMDMs (in the upper chamber) for 72 h. Ot-WHP (25, 50, 100 μg/ml) or vehicle (PBS) were added to macrophages in the upper chamber. After co-culture, macrophages in the upper chamber were discarded, and viable cells in the lower chamber were determined by a CCK-8 kit. The levels of collagen accumulation in the supernatant and α-SMA expression in fibroblasts in the lower chamber of the co-cultured system were determined by ELISA and Western blot, respectively. The average of 3 independent experiments is shown. ns, no significance, *p < 0.05, **p < 0.01.

To see if fibroblasts were effector cells of Ot-WHP, the direct effect of Ot-WHP on fibroblast cell proliferation was tested. As shown in Figure 12A, Ot-WHP did not have any effect on fibroblast cell proliferation at concentrations up to 100 μg/ml. Fibroblasts differentiate into a contractile phenotype, myofibroblasts, which is activated by many growth factors and cytokines in the wound sites, and fibroblast-to-myofibroblast transition is critical for wound contraction in cutaneous wound healing (46–48). Fibroblast-to-myofibroblast transition is characterized by the expression of α-smooth muscle actin (α-SMA). As shown in Figure 12B, Ot-WHP treatment significantly up-regulated the deposition of collagen from fibroblasts in the wound sites at day 8 relative to vehicle (PBS) treatment, and collagen-positive area in Ot-WHP-treated wounds was increased by 1.7-folds. In accordance with the collagen deposition, Ot-WHP treatment significantly up-regulated the expression of α-SMA in mouse cutaneous wound sites at day 8 as compared to vehicle (PBS) treatment, and α-SMA-positive area in Ot-WHP-treated wounds was increased by 1.2-folds (Figure 12C). We next investigated whether Ot-WHP directly induced α-SMA expression and collagen deposition in fibroblasts. As shown in Figures 13A,B, Ot-WHP had no significant effect on collagen deposition and α-SMA expression in fibroblasts without co-culture of BMDMs. The results suggested that Ot-WHP efficiently promoted fibroblast-to-myofibroblast transition and collagen production in mouse wound sites in an indirect manner.

### Ot-WHP Enhanced the Cross-Talk Between Macrophages and Keratinocytes/Fibroblasts

The healing of skin wound is a complex requiring the cross-talk of many cell types, including kinds of recruited inflammatory cells, and resident keratinocytes and fibroblasts (5). To investigate whether Ot-WHP enhanced the cross-talk between macrophages and keratinocytes/fibroblasts and to further understand how Ot-WHP induced fibroblast-to-myofibroblast transition in mouse wound sites, keratinocytes or fibroblasts were co-cultured with macrophages in the presence or absence of Ot-WHP. As shown in Figure 13C, the co-culture of keratinocytes with macrophages did not induce a significant proliferation of keratinocytes relative to cells without co-culture with macrophages, while the addition of Ot-WHP significantly induced the proliferation of keratinocytes in a dose-dependent manner. After the addition of 25, 50, and 100 μg/ml of Ot-WHP, the proliferation of keratinocytes was increased by 12.0, 26.2, and 41.1%, respectively. A similar result was observed in fibroblasts, and the proliferation of fibroblasts was increased by 5.0, 16.3, and 27.7% followed by co-culture with macrophages in the presence of 25, 50, and 100 μg/ml of Ot-WHP, respectively (Figure 13D). Similar results were also observed in α-SMA expression and collagen deposition in fibroblasts followed by co-culture with macrophages in the presence of Ot-WHP. As shown in Figures 13E,F, the co-culture of fibroblasts with macrophages did not induce a significant collagen deposition and α-SMA expression in fibroblasts relative to cells without co-culture of macrophages, while the addition of Ot-WHP significantly induced the collagen deposition and α-SMA expression in fibroblasts in a dose-dependent manner. After the addition of 25, 50, and 100 μg/ml of Ot-WHP, collagen deposition was increased by 10.5, 24.7, and 32.5%, and α-SMA expression was increased by 19.8, 36.7, and 66.5%, respectively. In combination with the data that Ot-WHP showed no direct effects on keratinocyte and fibroblast cell proliferation (Figures 11A, 12A), and showed no direct effects on collagen deposition and α-SMA expression in fibroblasts (Figures 13A,B), which suggested that Ot-WHP promoted the proliferation of keratinocytes and fibroblasts, collagen deposition and α-SMA expression in fibroblasts via activating macrophages. These results indicated that Ot-WHP significantly enhanced the crosstalk between macrophages and keratinocytes/fibroblasts, which in turn efficiently promoted wound closure in mice.

## Discussion

Wound healing is a cascade of events including inflammation, new tissue formation, and tissue remodeling (5, 49). After injury, inflammatory cells recruit to the wound site. Neutrophils arrive first within a few minutes, followed by monocytes and lymphocytes (49). The chemokines, cytokines, and growth factors secreted by inflammatory cells initiate the inflammatory phase of wound repair. Then keratinocytes and dermal fibroblasts migrate and proliferate at the wound edge. Furthermore, wound fibroblasts acquire a contractile phenotype and transform into myofibroblasts, a cell type which plays a major role in wound contraction. Before collagen matrix formation, granulation tissue acts as wound connective tissue. Theoretically speaking, regulation of inflammation, new tissue formation, and tissue remodeling are effective ways to promote wound healing. Up until now, wound healing therapies are mainly based on growth factors, which are large sizes that correspond to higher production cost and hardly generate an excellent prognosis in clinical application (10). Recently, immunomodulatory peptides with small molecular weight comprise a kind of excellent candidates for wound healing therapy (11, 12).

Over the past few decades, bioactive components especially bioactive peptides have been extensively studied in amphibian skin secretions (15, 50, 51). These peptides include antimicrobial peptides, opioid peptides, corticotropin-releasing peptides, angiotensins, protease inhibitor peptides, neuropeptides, antioxidant peptides, lectins, insulin-releasing peptides, wound-healing peptides, and immunomodulatory peptides and so on. They are stored in skin granular glands and can be released in high concentrations into skin secretions when frog is stressed or injured. The Chinese concave-eared frog *O. tormota* is an arboreal, nocturnal species living in the mountain area. Although *O. tormota* live in a harsh survival environment, their skin is still maintained in a healthy and balanced state, which implies that they have developed an effective defense system, including producing bioactive peptides. However, all the studies about *O. tormota* just focused on the investigation of the unique ultrasonic communication between male and female frogs (25–29), but there was no information about *O. tormota*-derived bioactive peptides. Considering their complicated habitats and the good condition of their skin, we presume that they have developed the capacity to regenerate and heal rapidly. As expected, we identified a bioactive peptide (Ot-WHP) with efficient wound healing-promoting activity derived from the skin of *O. tormota*.

In addition to Ot-WHP, a total of nine wound healing-promoting peptides have been characterized from amphibians (16–24). However, the mechanism of action of these peptides, including their effects on neutrophils, the direct effects or indirect effects of the peptides on effector cells (neutrophils, macrophages, keratinocytes and fibroblasts) and the effects of these peptides on the cross-talk between the effector cells, remained unclear (16–24). Therefore, it is still worth to further investigating the mechanism of action of Ot-WHP, which can provide new insights of amphibian-derived wound healing-promoting peptides.

Previous studies did not investigate the effect of amphibian-derived wound healing peptides on neutrophils in wound healing. In our study, Ot-WHP significantly increased the numbers of neutrophils in wound site at days 0.5 and 1 post injury (Figure 4). Neutrophils are a type of inflammatory cells which are the first to arrive to wound sites, clear off cell debris and invading pathogens, and secrete cytokines to serve local keratinocytes and fibroblasts (52). Our findings suggested that Ot-WHP modestly promoted neutrophil phagocytosis and PMA-induced NET formation, while Ot-WHP did not directly induce NET formation nor chemokine/cytokine production in neutrophils (Figure 5).

In addition, the macrophages play a critical regulatory role in the healing process, especially during the inflammatory phase, including initiation and the resolution of inflammatory processes (53). In the inflammatory phase of wound repair, macrophages produced several kinds of chemokines, cytokines and growth factors (53). Tissue macrophages do expand in wound healing, which critically modulates injury vs. healing (54). Although skin-resident macrophages are heterogeneous (55), BMDMs comprise an important type of macrophage, which are recruited to wounds and contribute to wound repair (56). As an excellent model to study various mechanisms in a primary cell culture, BMDMs were selected to investigate the effects of Ot-WHP on macrophages in our study, which at least partly reveals the regulatory effects of Ot-WHP on macrophages in wound healing. Our data indicated that Ot-WHP significantly increased the number of macrophages in wound sites at days 2 and 3 post injury (Figure 6); and Ot-WHP directly enhanced the production of chemokines, cytokines, and growth factor in mouse BMDMs and mouse wound sites (Figure 7); and Ot-WHP exerted these regulatory effects on BMDMs via activating MAPKs, NF-κB, and Smad signaling pathways. These findings were also described in previous reports of amphibian-derived wound healing peptides.

Previous studies confirmed that amphibian-derived wound healing peptides increased the numbers of inflammatory cells (macrophages) in wounds. However, these studies did not investigate whether these peptides could directly induce neutrophil and macrophage migration. In our study, we confirmed that Ot-WHP could not act as a chemoattractant for neutrophils and macrophages, despite the increase in neutrophils and macrophages at the wounds by Ot-WHP. Interestingly, Ot-WHP markedly enhanced neutrophil and macrophage migration in the presence of macrophages (Figures 8B,D), suggesting that the chemotactic activity of Ot-WHP depends on inducing chemoattractant production in macrophages.

As mentioned above, Ot-WHP directly induced the production of regulatory factors in macrophages, which implied that Ot-WHP possibly activated macrophages at the first moment post injury, consequently strengthened the interplay between different types of effector cells in wound healing, and finally promoted wound healing. As expected, Ot-WHP significantly enhanced the proliferation of keratinocytes or fibroblasts, and significantly promoted α-SMA and collagen deposition in fibroblasts in the co-culture of macrophages (Figure 13), indicating that Ot-WHP efficiently promoted the cross-talk between macrophages and keratinocytes or fibroblasts. This finding is a novel advancement of the effects of amphibian-derived wound healing peptides on the crosstalk between different effector cell types.

If neutrophils and macrophages constantly infiltrate, it will lead to the over-expression of chemokines, cytokines, growth factors and proteases (i.e., matrix metalloproteases 2, 8, and 9), and in turn result in sustained inflammation and chronic wounds (57–59). Ot-WHP significantly enhanced the recruitment of neutrophils to wound sites on days 0.5 and 1 post injury (Figure 4), and significantly enhanced the recruitment of macrophages to wound sites on days 2 and 3 post injury (Figure 6). Nevertheless, neutrophil recruitment enhanced by Ot-WHP at day 1 showed a declining trend relative to day 0.5 (Figure 4). Meanwhile, the chemokines, cytokines and growth factors induced by Ot-WHP in wound sites also decreased at days 4 and 6 post injury (Figure 7). Most importantly, the wounds that received Ot-WHP treatment showed a better prognosis as compared to vehicle-treated wounds. Therefore, it is more likely that Ot-WHP efficiently promoted wound healing by initiating and accelerating the inflammation in early inflammatory phase rather than by prolonging inflammatory phase, which highlights the potential of Ot-WHP for management of acute wound healing.

Ot-WHP shows some sequence overlap with AH90 (about 83% identity). Accordingly, we hypothesize that Ot-WHP probably exhibits some similar mechanisms to Ot-WHP in wound healing. As expected, several findings about the mechanism of Ot-WHP in wound healing are confirmatory of those of AH90. These confirmatory findings include: they both induced the production of regulatory factors in macrophages via activating MAPKs and NF-κB signaling pathways; both activated TGF-β-dependent Smad signaling pathway in macrophages; and both enhanced the migration of keratinocytes by promoting cell adhesion and integrin expression. In addition, Ot-WHP increased the numbers of neutrophils in wound sites and promoted neutrophil phagocytosis and PMA-induced neutrophil extracellular trap formation; Ot-WHP increased the numbers of macrophages and the production of regulatory factors in wound sites; Ot-WHP did not act as a chemoattractant for neutrophils or BMDMs alone, but it was chemotactic to neutrophils and BMDMs in co-culture with macrophages; Ot-WHP promoted fibroblast-to-myofibroblast transition and collagen deposition in wound sites; Ot-WHP enhanced the cross-talk between macrophages and keratinocytes/fibroblasts. These findings were not investigated in AH90, which provided new insights into the mechanism of action of amphibian-derived wound healing-promoting peptides.

Compared to EGF, Ot-WHP exhibited a unique mechanism in promoting wound healing. Ot-WHP recruited more neutrophils and macrophages to wounds via eliciting the production of regulatory factors in macrophages, which resulted in the initiation and acceleration of the inflammatory phase. In addition, Ot-WHP promoted the proliferation of keratinocytes and fibroblasts, and collagen deposition and α-SMA expression in fibroblasts via enhancing the crosstalk between macrophages and keratinocytes/fibroblasts. Whereas, EGF did not affect the inflammatory phase in the process of wound healing. But EGF directly promoted the proliferation of keratinocytes and fibroblasts *in vitro* (Figure S3) (60, 61), and collagen deposition and α-SMA expression in wounds (62).

In conclusion, an *O. tormota*-derived peptide, Ot-WHP, efficiently promoted cutaneous wound healing in mice. Ot-WHP initiated and accelerated the inflammatory phase by recruiting neutrophils and macrophages to wound sites post injury. Ot-WHP modestly regulated neutrophil phagocytosis, and directly acted on macrophages by inducing the production of chemokines, cytokines, and growth factor. Besides, keratinocytes and fibroblasts constituted another two important effector cell types of Ot-WHP. Ot-WHP directly promoted keratinocyte migration by up-regulating of integrin expression, but it had no direct effect on keratinocyte or fibroblast proliferation, or fibroblast differentiation. However, Ot-WHP significantly promoted keratinocyte proliferation, fibroblast proliferation, and fibroblast differentiation in the presence of macrophages, which indicated that Ot-WHP enhanced the cross-talk between macrophages and keratinocytes/fibroblasts. Our results provided new insight into the mechanism of action of amphibian-derived wound healing peptide and provided a potential peptide immunomodulator for acute wound healing therapy.

## Data Availability Statement

The datasets generated for this study can be found in the GenBank accession number, MK431780 (https://www.ncbi.nlm.nih.gov/nuccore/MK431780).

## Ethics Statement

The animal study was reviewed and approved by Animal Care and Use Committee as well as the Ethical Committee of Soochow University.

## Author Contributions

XH, YY, LM, and YZ contributed to experimental studies and data analysis. YC and JW contributed to the data analysis. XH and LW wrote the manuscript. YW, HY, and ML played a major role in animal experiments. WX and LW contributed to financial support and gave final approval for publication of the manuscript.

### Conflict of Interest

The authors declare that the research was conducted in the absence of any commercial or financial relationships that could be construed as a potential conflict of interest.

## Abbreviations

EGF: epidermal growth factor
FGF-2: fibroblast growth factor 2
VEGF: vascular endothelial growth factor
PDGF: platelet-derived growth factor
KGF-1: keratinocyte growth factor-1
GM-CSF: granulocyte macrophage colony-stimulating factor
G-CSF: granulocyte colony-stimulating factor
BMDMs: Bone marrow-derived macrophages
H&E: hematoxylin and eosin
NETs: neutrophil extracellular traps
ELISA: Enzyme-linked immunosorbent assay
PMA: phorbol myristate acetate
α-SMA: α-smooth muscle actin.
