# Supplementary material for: A Frog-Derived Immunomodulatory Peptide Promotes Cutaneous Wound Healing by Regulating Cellular Response
Source: Front Immunol. 2019 Oct 17;10:2421. doi: 10.3389/fimmu.2019.02421 (PMC6812695; doi:10.3389/fimmu.2019.02421)
Supplement: Supplementary file 1 [file Data_Sheet_1.docx]

**A frog-derived immunomodulatory peptide promotes cutaneous wound healing by regulating cellular response**

Xiaoqin He*^,†,1^, Yang Yang*^,1^, Lixian Mu^‡,1^, Yandong Zhou*^,1^, Yue Chen*, Jing Wu^‡^, Yipeng Wang^§^, Hailong Yang^‡^, Min Li*, Wei Xu*^,2^ and Lin Wei*^,2^

*Jiangsu Key Laboratory of Infection and Immunity, Institutes of Biology and Medical Sciences, Soochow University, Suzhou 215123, Jiangsu, China;

**^†^**Key Laboratory of National Health and Family Planning Commission on Parasitic Disease Control and Prevention, Jiangsu Provincial Key Laboratory on Parasite and Vector Control Technology, Jiangsu Institute of Parasitic Diseases, Wuxi 214064, Jiangsu Province, China;

**^‡^**School of Basic Medical Sciences, Kunming Medical University, Kunming 650500, Yunnan, China;

**^§^**Department of Pharmaceutical Sciences, College of Pharmaceutical Sciences, Soochow University, Suzhou, Jiangsu 215123, China.

**Supplemental figures**


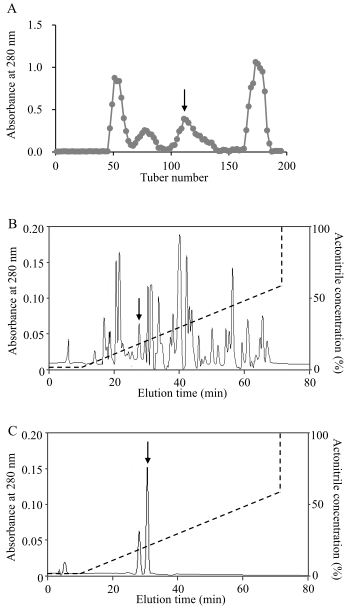


**Figure S1. Purification of Ot-WHP from the skin secretions of *O. tormotus.*** (A) Sephadex G-50 gel filtration**.** The skin secretions were applied to Sephadex G-50 gel filtration column and eluted with 0.1M PBS in a flow rate of 3 ml/10 min, and the fraction containing wound healing-promoting activity is marked by an arrow. (B) The interesting fraction from the Sephadex G-50 gel filtration was purified by a C18 RP-HPLC column with the indicated gradient of acetonitrile in 0.1% (v/v) trifluoroacetic acid in water, and the fraction containing wound healing-promoting activity is marked by an arrow. (C) The eluted peak (arrowed in B) was further purified by a C18 RP-HPLC column. The purified Ot-WHP is indicated by an arrow*.*


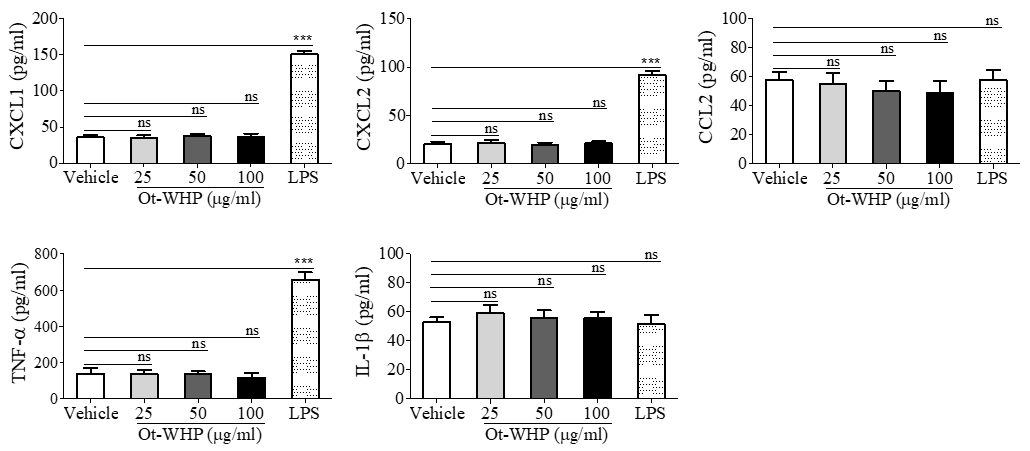


**Figure S2. Effect of Ot-WHP on the production of chemokines and cytokines in neutrophils.** Ot-WHP (25, 50, 100 μg/ml), an equal volume of vehicle (PBS) or LPS (100 ng/ml) was incubated with neutrophils (5×10^5^ cells/well, 24-well plate) at 37℃ for 4 h in RPMI 1640 supplemented with 2% FBS. The levels of chemokines (CXCL1, CXCL2 and CCL2) and cytokines (TNF-α and IL-1β) in supernatants were tested by ELISA. ns, no significance, ****p* < 0.001.


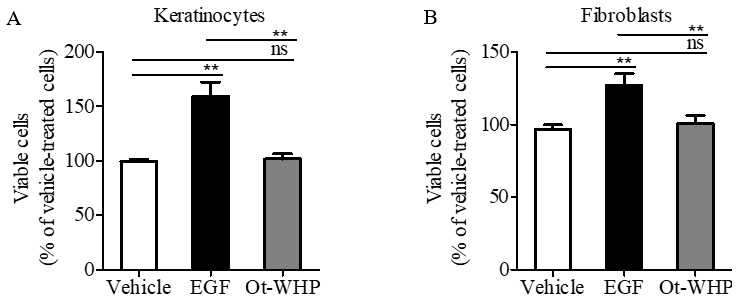


**Figure S3. EGF efficiently promoted the proliferation of keratinocytes and fibroblasts.** Keratinocytes (HaCat) or fibroblasts (isolated from newborn mice skin) (10^4^ cells/well, 100 μl/well) were cultured with DMEM supplemented with 2% FBS in 96-well culture plates. After the addition of EGF (10 ng/ml), Ot-WHP (100 μg/ml, control), or an equal volume of vehicle (PBS), keratinocytes were cultured for 24 h, and fibroblasts were cultured for 72 h. Then, cell proliferation was detected by a CCK-8 kit. ns, no significance, ***p* < 0.01.
